# Lipid Regulation Effects of Raw and Processed Notoginseng Radix Et Rhizome on Steatotic Hepatocyte L02 Cell

**DOI:** 10.1155/2016/2919034

**Published:** 2016-08-24

**Authors:** Zhu Chen, Chunmei Li, Caixia Yang, Ronghua Zhao, Xiaojian Mao, Jie Yu

**Affiliations:** Yunnan University of Traditional Chinese Medicine, Kunming, Yunnan 650500, China

## Abstract

*Introduction*. Raw and processed Notoginseng Radix Et Rhizome (NRR) have been widely used in treatment of metabolic syndromes and related disease, including nonalcoholic fatty liver disease (NAFLD). This study was designed to investigate lipid regulation effects of raw and processed NRR in steatotic L02 cell.* Materials and Methods*. Steatotic L02 cells were obtained after being cultured with 5% fat emulsion-10% FBS-RPMI 1640 medium for 48 h. Contents of total cholesterol (TC), triglyceride (TG), free fatty acid (FFA), high-density lipoprotein cholesterol (HDL-C), and low-density lipoprotein cholesterol (LDL-C) in steatotic L02 cells were evaluated after treatment. Furthermore, the lipid metabolism regulation mechanism of* Panax notoginseng* saponins (PNS) and its monomers were evaluated by detecting the expressions of hydroxymethyl glutaric acyl coenzyme A reductase (HMG-CoAR), sterol regulating element binding protein-2 (SREBP-2), and cholesterol 7*α*-hydroxylase (CYP7*α*).* Results*. TG and TC contents were doubled in model group compared to those in normal L02 cells group. Raw NRR and NRR heated with sand (NRR-B) showed much remarkable lipid-lowering effects in steatotic L02 cells. PNS, notoginsenoside R_1_, ginsenoside Rg_1_, and ginsenoside Rb_1_ displayed the best TG and TC regulation activity, which could significantly reduce contents of SREBP-2 and HMG-CoAR and increase the content of CYP7*α*.* Conclusions*. Our results may support the fact that both raw NRR and NRR-B might have more satisfactory effects in the treatment of NAFLD.

## 1. Introduction

Nonalcoholic fatty liver disease (NAFLD) is a common health problem worldwide. As the data collected from all over the world shows, about one in four adults suffers from NAFLD. In western and Asian countries, prevalence of NAFLD is up to approximately 30% in the general population. The detection rate of NAFLD increased with age and body mass index (BMI) [1]. The prevalence of NAFLD was increased from 35.47% in 2006 to 46.46% in 2014 among 1948 subjects in China [2]. NAFLD, highly related to metabolic syndrome, is defined as the complex lipid metabolism dysfunction in hepatic cells. Hypertriglyceridemia (HTG), which may or may not be associated with hypercholesterolemia, is usually presented in NAFLD. Insulin resistance and inflammation are believed to play main roles in the pathogenesis and progression of NAFLD. Metabolic syndromes, which include obesity, type 2 diabetes mellitus, and hyperlipidemia, are risk factors of NAFLD [3].

Notoginseng Radix Et Rhizome (NRR), known as a valuable traditional Chinese medicine, is the dried roots of* Panax notoginseng* (Burk.) F. H. Chen in Araliaceae family. NRR can be used with its both raw and processed products. Several processing methods are recorded and applied in clinic use of NRR. NRR can be steamed solely [4, 5] or heated with sand or sesame oil [6, 7]. Different processed products of NRR have obvious differences in clinical practice application.

*Panax notoginseng* has got a high evaluation in the treatment of hematic disease, such as coronary artery disease, angina, peptic ulcer, hyperlipidemia, and hypertension [8, 9]. Raw NRR could promote blood circulation for removing blood stasis, remove edema, and relieve pain [10]. It is considered as sovereign remedies for traumatism and first-line choice for hemostasis in traditional Chinese medicine. The steamed NRR (NRR-A) and NRR heated with sand (NRR-B) have better pharmacological effects in nourishing blood and improving the microcirculation [11]. NRR fried with sesame oil (NRR-C) plays a major role in enhancing memory retention and the immunity of the body [12]. In recent decades, raw and processed NRR are all used in the treatment of NAFLD and metabolic syndromes related disease [13]. The contents of* Panax notoginseng* saponin (PNS, which is one of the most important compounds extracted from roots of the herb* Panax notoginseng*) and its monomers, such as notoginsenoside R_1_, ginsenoside Rg_1_, ginsenoside Rb_1_, ginsenoside Re, and ginsenoside Rd, reduced in different degrees after processing [14, 15]. However, little is known about the activities and possible differences between raw and processed NRR on lipid regulation.

In this study, steatotic L02 hepatocyte of normal human was used to explore the lipid metabolism regulation effects of raw NRR and its processed products. Moreover, potential activities of PNS, notoginsenoside R_1_, ginsenoside Rg_1_, ginsenoside Rb_1_, ginsenoside Rd, and ginsenoside Re were also investigated.

## 2. Materials and Methods

### 2.1. Main Reagents

PNS (the contents of notoginsenoside R_1_, ginsenoside Rg_1_, ginsenoside Rb_1_, ginsenoside Rd, and ginsenoside Re are 6.9%, 28.0%, 29.7%, 7.3%, and 3.8%, resp.), notoginsenoside R_1_, ginsenoside Rg_1_, ginsenoside Rb_1_, ginsenoside Rd, and ginsenoside Re were purchased from the National Institute for the Control of Pharmaceutical and Biological Products, China. Lovastatin (Jiangsu Ocean Pharmaceutical Co., Ltd., China) and fenofibrate (Laboratoires Fournier S.A., France) were used as positive control for lowering cholesterol and triglyceride, respectively. Fetal bovine serum (FBS) and penicillin-streptomycin were purchased from Fisher Scientific International Inc., USA. Fat emulsion was purchased from Sichuan Koren Pharmaceutical Co., Ltd., China.

### 2.2. Processing Procedures of NRR

**Figure 1:**
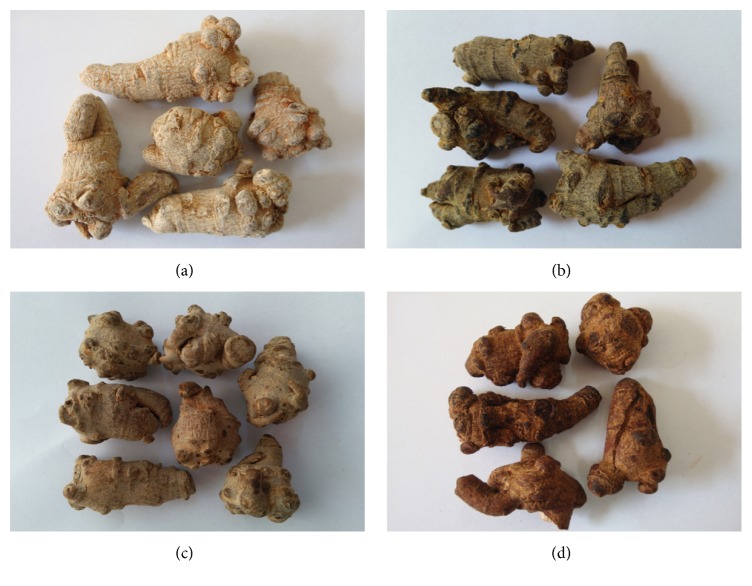
Photographs of Notoginseng Radix Et Rhizome and its processed products: (a) NRR, (b) NRR-A, (c) NRR-B, and (d) NRR-C.

The Notoginseng Radix Et Rhizome was collected in Wenshan County of Yunnan Province by the authors in September 2012 and identified as the root and rhizome of* P. notoginseng* (Burk.) F. H. Chen by Professor Xiaojian Mao, Yunnan University of Traditional Chinese Medicine. Voucher specimens were deposited in the Herbarium of Pharmacognosy, Yunnan University of Traditional Chinese Medicine. NRR-A was steamed from NRR solely at 180°C in a high pressure cooker for 6 h. NRR-B was heated from NRR with sand (mineral or rock grains with the particle size of 0.074~2 mm) at about 90~100°C for 90 minutes, while NRR-C was fried from NRR with sesame oil at about 90~100°C for 90 minutes. These processing procedures of NRR-A and NRR-C were executed strictly according to the procedures recorded in Chinese Pharmacopoeia, 2015 edition. The processing procedure of NRR-B was drawn on the records in Modern Research in Processing Chinese Herbs [16]. NRR, NRR-A, NRR-B, and NRR-C used in this research were shown in Figure 1.

### 2.3. Determination of Notoginsenoside R_1_, Ginsenoside Rg_1_, Ginsenoside Rb_1_, Ginsenoside Rd, and Ginsenoside Re Contents in the Raw and Processed NRR by HPLC

In order to illustrate the chemical profiles of raw and processed NRR, concentrations of notoginsenoside R_1_, ginsenoside Rg_1_, ginsenoside Rb_1_, ginsenoside Re, and ginsenoside Rd were detected by HPLC. Sample solutions were prepared following the procedures developed in our previous studies [14]. 0.6 g powder of NRR, NRR-A, NRR-B, and NRR-C was precisely weighed and refluxed with 50 mL 70% methanol for 2 h. These solutions were filtered through 0.45 *μ*m membrane and 10 *μ*L was injected into the HPLC system, respectively.

All experiments were performed with Agilent Zorbax SB-C18 (4.6 mm × 250 mm, 5 *μ*m). The mobile phase consisted of acetonitrile (A) and water (B) was used as gradient elution for separation. The following gradient was used: 0–21 min, 20% A; 21–30 min, 20–25% A; 30–41 min, 25–41% A; 41–51 min, 41–45% A; 51–56 min, 45–20% A; and 56–66 min, 20% A. The column temperature was maintained at 20°C. The analysis was conducted at a flow rate of 1.0 mL/min and detected at 203 nm.

### 2.4. Preparation of Extractions of Raw and Processed NRR

10 g powdered samples of raw and processed NRR were refluxed with 100 mL 50% ethanol for three times, 1 hour for each time, separately. The extracts were combined, condensed, and lyophilized. The extraction rates of NRR, NRR-A, NRR-B, and NRR-C were 18.0%, 16.5%, 19.0%, and 16.0% crude drug, respectively.

### 2.5. Culture Conditions and Treatment of Cells [17]

The whole research was carried out by human normal hepatocyte cell line L02, which was purchased from Kunming Institute of Zoology, Chinese Academy of Sciences. The cell was maintained in an incubator (MCO-20AIC CO_2_ Incubator, Japan's Sanyo Electric Co., Ltd.) with 5% carbon dioxide, 95% air, at 37°C in RPMI-1640 medium (Gibco Invitrogen Corporation, USA) supplemented with 10% fetal bovine serum (FBS, Hyclone). 0.25% trypsin (1~2 mL) (Amresco, USA) was used to passage cells at 80~90% confluence.

L02 cells were inoculated into 6-well plates at a cell density of 3 × 10^5^ and then incubated for 48 h till 80~90% confluences with 10% FBS-RPMI 1640 medium. Cells were incubated in G_0_ medium (0.2% FBS-RPMI 1640 medium) for 24 h to induce the cell cycle synchronous before the test. In the subsequent experiments, the control group was incubated with 10% FBS-RPMI 1640 medium alone, while the model group was incubated with 5% fat emulsion-10% FBS-RPMI 1640 medium for 48 h, respectively. After 48 h, cell morphological changes were investigated by inversion fluorescence microscope (ECLIPSE TS100, Nikon Corporation, Japan).

### 2.6. Preparation of Treatment Solutions

The stock solutions of raw and processed NRR extracts were achieved by dissolving them in dimethylsulfoxide (DMSO, Sigma, USA) at a concentration of 10 mg/mL. Then they were further diluted with G_0_ medium (0.2% FBS-RPMI 1640 medium) to grade concentrations of 10 *μ*g/mL, 20 *μ*g/mL, 40 *μ*g/mL, 80 *μ*g/mL, and 100 *μ*g/mL, respectively.

Similarly, the* Panax notoginseng* saponins were diluted with G_0_ medium to grade concentrations of 10 *μ*g/mL, 20 *μ*g/mL, and 40 *μ*g/mL, respectively. Meanwhile, lovastatin and fenofibrate were diluted with G_0_ medium to make stock solutions at a concentration of 10 *μ*g/mL.

### 2.7. Lipid Metabolism Regulation Effects

Steatotic hepatocyte L02 cells were treated with the extracts of NRR, NRR-A, NRR-B, and NRR-C at the concentrations of 10, 20, 40, 80, and 100 *μ*g/mL for another 24 h to evaluate their lipid metabolism regulation activity. 24 h after exposure, the cells were washed with PBS for 2 times. Then, the cells were trypsinized, collected, and resuspended with PBS and centrifuged to separate PBS from cells. Cells debris were lysed by 0.01% Triton X-100 (Sigma, USA) solution and then centrifuged at 5,000 rpm (Allegra X-22 Centrifuge) (Beckman Coulter, USA) for 10 minutes. TG and TC contents in the supernatant were examined using assay kits purchased from the Biosino Bio-technology & Science Inc. The contents of HDL-C and LDL-C in the supernatant were tested by assay kits purchased from the Changchun Sunostik Medical Technology Co., Ltd., China. FFA contents in the supernatant were tested by assay kits purchased from the Nanjing technology Co., Ltd., China. All these assays were carried out by DNM-9602G enzyme microplate reader purchased from the Beijing Pulang New Technology Co., Ltd., China.

Cells were lysed and collected under the above conditions. The contents of TG and TC in PNS, Rg_1_, Rb_1_, Rd, Re, and R_1_ treated groups at the concentrations of 10, 20, and 40 *μ*g/mL were measured by DNM-9602G enzyme microplate reader.

In order to explore the mechanism of* Panax notoginseng* on lipid metabolism, the contents of cholesterol 7 alpha hydroxylase, HMG-CoAR, and SREBP-2 in PNS, R_1_, Rg_1_, and Rb_1_ treated groups were tested by ELISA assay kits purchased from Wuhan biological engineering Co., Ltd., China.

### 2.8. Statistical Analysis

Statistical analysis was performed using SPSS version 17. The quantitative data were given as the mean ± SD. The data of multiple groups were compared with one-way analysis of variance. *p* values < 0.05 were considered significant.

## 3. Results

### 3.1. The Contents of Triterpenoid Saponins in Raw and Processed NRR

The percentages of notoginsenoside R_1_, ginsenoside Rg_1_, ginsenoside Rb_1_, ginsenoside Re, and ginsenoside Rd in raw and processed NRR were listed in Table 1. Most of the saponins contents reduced after processing. The contents of ginsenoside Rg_1_, ginsenoside Rb_1_, ginsenoside Re, and ginsenoside Rd in processed NRR appear to be lower than that in raw NRR, but statistical significance for such difference was not observed. The degradations of notoginsenoside R_1_, ginsenoside Rg_1_, ginsenoside Rb_1_, ginsenoside Re, and ginsenoside Rd were most dramatically in NRR-B. However, percentage of notoginsenoside R_1_ was increased in NRR-C.

### 3.2. Fat Emulsion Incubation Induced Cellular Steatotic

A model of steatotic hepatocyte was successfully established by the incubation with 5% fat emulsion-10% FBS-RPMI 1640 medium in human liver L02 cell line. Morphological changes indicated that lipid droplets (LDs) were obviously accumulated in the model group. Cell swelling, characteristic of cell death and cell gap, grew bigger than that of the control group. As the positive control, only slight dead cells were observed, with no significant accumulation of LDs. The intracellular TG contents were increased from 0.25 ± 0.01 mmol/L to 0.53 ± 0.01 mmol/L (*p* < 0.001) in steatotic L02 cells, while the intracellular contents of TC were increased from 0.13 ± 0.01 mmol/L to 0.23 ± 0.01 mmol/L (*p* < 0.001), which indicated the full and successful simulation of human NAFLD status.

### 3.3. TG, TC, HDL-C, LDL-C, and FFA Regulation Activity of Raw and Processed NRR

The TG, TC, HDL-C, LDL-C, and FFA contents in raw and processed NRR were shown in Tables 2, 3, 4, 5, and 6. Morphological observations showed that raw and processed NRR could reduce the accumulation of LDs at different levels, cells arranged closely, and intercellular space is small. The morphological changes of NRR-B were similar to the positive control groups. 50% ethanol extract of NRR and NRR-B showed better TG and TC regulation activity than NRR-A and NRR-C. Nevertheless, the effects of them were weaker than those of lovastatin and fenofibrate. 50% ethanol extract of NRR could reduce TC content by about 43% (*p* < 0.001), and 50% ethanol extract of NRR-B reduced TG content by about 24% (*p* < 0.001). HDL-C content was increased from 0.22 ± 0.03 mmol/L to 0.41 ± 0.02 mmol/L in 10 *μ*g/mL NRR-B group (*p* < 0.01), which was similar to that of control group. 50% ethanol extract of NRR and NRR-B could reduce LDC-L content by 47% and 39% (*p* < 0.001), respectively. 50% ethanol extract of NRR showed more remarkable FFA regulation effects compared to its processed procedures, and the FFA content in 100 *μ*g/mL NRR group was reduced from 762.89 ± 52.31 mmol/L to 386.59 ± 15.46 mmol/L in steatotic L02 cells (*p* < 0.001).

### 3.4. TG and TC Regulation Activities of Triterpenoid Saponins

**Figure 2:**
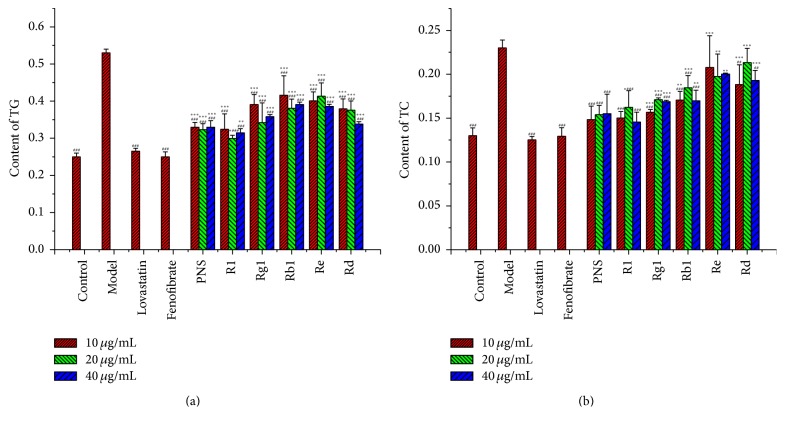
TG and TC contents in the steatotic L02 cells after treatment of triterpenoid saponins. Triglyceride (TG) (a) and total cholesterol (TC) (b) contents were assayed by assay kits as described in the text. Values were mean ± SD (*n* = 3) and expressed in mmol/L. NRR, Notoginseng Radix Et Rhizome; PNS,* Panax* notoginsenosides. *∗* indicates a significant difference compared with control group cells, and # indicates a significant difference compared with model group cells. ^*∗∗*^ *p* < 0.01. ^*∗∗∗*^ *p* < 0.001. ^##^ *p* < 0.01. ^###^ *p* < 0.001.

PNS, including but not limited to notoginsenoside R_1_, ginsenoside Rg_1_, ginsenoside Rb_1_, ginsenoside Re, and ginsenoside Rd, represented the main active ingredients of* P. notoginseng*. Changes in cell morphology indicated that PNS and notoginsenoside R_1_ could significantly reduce the formation of LDs, and the arrangement of basal epithelial cells was tight. The TG-lowering and TC-lowering effects of PNS and related triterpenoid saponins were displayed in Figures 2(a) and 2(b). PNS, notoginsenoside R_1_ and ginsenoside Rg_1_, showed better TG-lowering activities than those of ginsenoside Rb_1_, ginsenoside Re, and ginsenoside Rd. 40 *μ*g/mL ginsenoside Rg_1_ group could reduce the TG content from 0.53 ± 0.01 to 0.31 ± 0.01 mmol/L (*p* < 0.001), which was similar to that of lovastatin. PNS and notoginsenoside R_1_ could reduce about 35% of TC content (*p* < 0.001), while ginsenoside Rg_1_ and ginsenoside Rb_1_ could reduce about 26% of TC content (*p* < 0.001).

### 3.5. The Mechanism of* Panax notoginseng* on Lipid Metabolism

**Figure 3:**
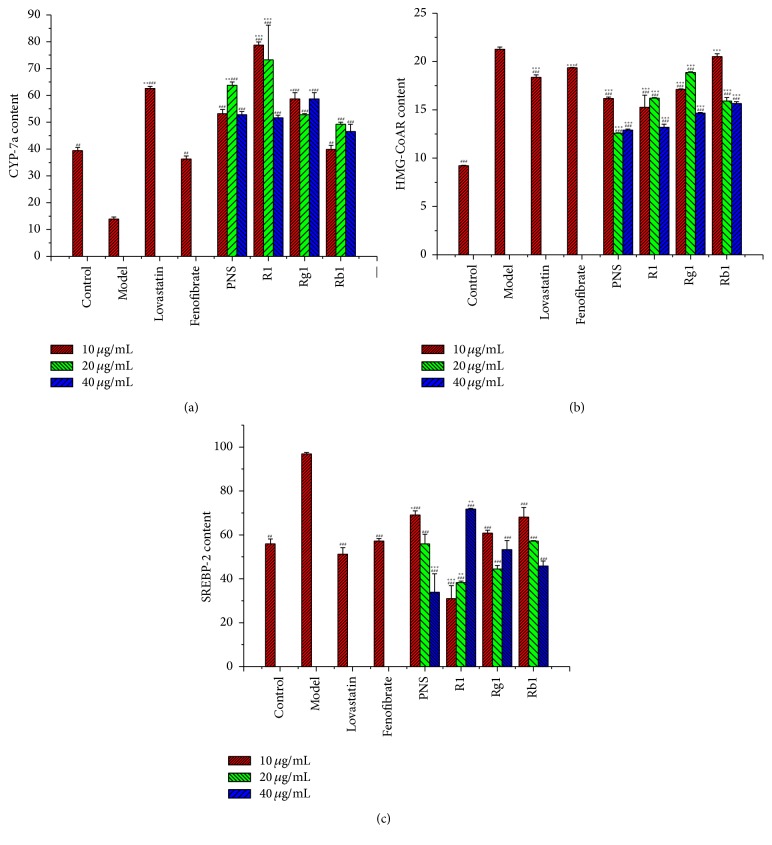
CYP7*α*, HMG-CoAR, and SREBP-2 contents in the steatotic L02 cells after treatment of triterpenoid saponins. CYP7*α* (a), HMG-CoAR (b), and SREBP-2 (c) contents were assayed by assay kits as described in the text. Values were mean ± SD (*n* = 3) and expressed in mmol/L. PNS,* Panax* notoginsenosides. *∗* indicates a significant difference compared with control group cells, and # indicates a significant difference compared with model group cells. ^*∗*^ *p* < 0.05. ^*∗∗*^ *p* < 0.01. ^*∗∗∗*^ *p* < 0.001. ^##^ *p* < 0.01. ^###^ *p* < 0.001.

Lipid-regulating effects are associated with the increasing of CYP7*α* and decreasing of SREBP-2 content, suggesting a possible antisteatotic mechanism of PNS, notoginsenoside R_1_, ginsenoside Rg_1_, and ginsenoside Rb_1_. The relative contents of CYP7*α*, HMG-CoAR, and SREBP-2 after treatments were shown in Figures 3(a), 3(b), and 3(c). Notoginsenoside R_1_ at the concentration of 10 *μ*g/mL showed great CYP7*α* increasing effect from 13.87 ± 0.77 to 78.73 ± 1.18 mmol/L (*p* < 0.001). PNS and notoginsenoside R_1_ could relieve the steatotic state of liver by lowering the HMG-CoAR content from 21.88 ± 0.23 to 12.91 ± 0.09 and 13.19 ± 0.33 mmol/L at the concentration of 10 *μ*g/mL (*p* < 0.001), respectively. The SREBP-2 regulation activities of high concentration group of PNS and low and medium concentration groups of notoginsenoside R_1_ showed a better effect than that of the positive group. They reduced nearly 65% of SREBP-2 content (*p* < 0.001). SREBP-2 lowering effects of ginsenoside Rg_1_ and ginsenoside Rb_1_ were weaker than those of other groups.

## 4. Discussion

TG and TC contents in hepatocytes are the defining characteristic of NAFLD. In addition, FFA has a direct effect on NAFLD when it esterified to TG [18]. LDL is in charge of transporting most of the lipid synthesized in the liver to the whole body via circulation system. On the contrary, HDL is in charge of transporting lipid back to the liver for biodegradation. Thus, it is important to point out that the levels of TG, TC, HDL-C, LDL-C, and FFA were the key factors for hyperlipidemia. All of them are considered as typical indicators in the diagnosis of NAFLD, hypertriglyceridemia, and hypercholesterolemia.

CYP7*α* and HMG-CoAR are key enzymes of biosynthesis and biodegradation of TC, while these procedures are regulated by SREBP-2. HMG-CoAR is the rate-limiting enzyme for cholesterol synthesis. Inhibiting its activity could reduce the LDL-C synthesis. CYP7*α* is the key rate-limiting enzyme for cholesterol transformation into bile acid in the liver, therefore promoting the excretion of cholesterol [19]. SREBP-2 is an important transcription factor, which could regulate fatty acid and cholesterol synthesis and impact the transcription of several genes involved in fatty acid and triglyceride metabolism [20].

In this research, L02 hepatocyte of normal human was exposed to 10% FBS and 5% fat emulsion for 48 hours as study objects. Steatotic human liver L02 cell was considered as a rapid and sensitive* in vitro* model to investigate the lipid regulation effects of raw and processed NRR. The contents of TG and TC in model group increased drastically compared to the control group (*p* < 0.001). The model can provide a reliable model for experimental study of pathogenesis of NAFLD and screening drugs for NAFLD treatment. Therefore, this steatotic model was frequently used in published investigations by our research group [21] and other scientists [22].

Processing procedure changed both chemical constituents and pharmacological activities of NRR. Data suggest that 50% ethanol extracts of NRR and NRR-B displayed distinctive lipid-lowering effects in steatotic L02 cells. NRR-A showed ordinary lipid-lowering effects, while NRR-C fared the worst.

Saponins, dencichine, volatile oil, and polysaccharides are the main phytochemical constituents in NRR. Among them, saponins are considered to be one of the most important active ingredients. Notoginsenoside R_1_, ginsenoside Rg_1_, and ginsenoside Rb_1_ were high abundance compositions [23] among all saponins with distinctive TG-lowering and TC-lowering activities. On the whole, notoginsenoside R_1_, ginsenoside Rg_1_, and ginsenoside Rb_1_ showed the best TG-lowering activities similar to that of PNS in the concentration of 40 *μ*g/mL in steatotic L02 cells compared with other triterpenoid saponins.

The contents of triterpenoid saponins decreased after processing, possibly due to the high temperature during processing procedure. Contents of triterpenoid saponins decreased most significantly in NRR-B; however, NRR-B showed the best activity on alleviating the fat denaturation, and that is probably related to the generation of other new constituents in the processing procedure [14]. Therefore, PNS, R_1_, Rg_1_, and Rb_1_, but not limited to them, were considered as material foundations of* Panax notoginseng*.

Abnormal cholesterol level in steatotic L02 cells could be associated with the cholesterol biosynthesis pathway and the transformation-related protein expression. PNS, Rg_1_, Rb_1_, and R_1_ could inhibit cholesterol synthesis and reduce the content of SREBP-2. PNS, Rg_1_, Rb_1_, and R_1_ showed better lipid-lowering activities by increasing the content of CYP7*α* and downregulated the TC content in steatotic L02 cells. The study of* Panax notoginseng* on lipid-lowering and its mechanisms can provide new ideas for the further studies on NAFLD.

In conclusion, raw NRR and NRR heated with sand show better lipid regulation effects than other processing procedures in the treatment of hyperlipidemia, NAFLD, and its related diseases. PNS, notoginsenoside R_1_, ginsenoside Rg_1_, ginsenoside Rb_1_, and ginsenoside Rd may be the main active ingredients to reduce TG and TC. The mechanisms of lipid-lowing effect of PNS, notoginsenoside R_1_, ginsenoside Rg_1_, and ginsenoside Rb_1_ may be related to the downregulation of HMG-CoAR and SREBP-2 and upregulation of CYP7*α*. Future studies focusing on animal experiments are still being carried out by our research group in order to elucidate the lipid regulation activities of raw and processed NRR* in vivo*.

## Supplementary Material

The results of L02 cellular morphology by the inverted microscope were displayed in figure 1. 2. 3, and the lipid droplets accumulation in model group were more significantly than that in normal and positive group. Different concentrations of Raw and processed NRR and its triterpenoid saponins have different effects on cell morphology.

## Figures and Tables

**Table 1:** The percentage of notoginsenoside R_1_, ginsenoside Rg_1_, ginsenoside Re, ginsenoside Rb_1_, and ginsenoside Rd in Notoginseng Radix Et Rhizome and its processed products.

Sample	Notoginsenoside R_1_	Ginsenoside Rg_1_	Ginsenoside Re	Ginsenoside Rb_1_	Ginsenoside Rd
NRR	0.98	3.27	0.38	2.72	0.66
NRR-A	0.79	2.69	0.33	2.49	0.61
NRR-B	0.30	1.15	0.11	1.00	0.24
NRR-C	1.09	2.56	0.18	2.44	0.57

The contents of notoginsenoside R_1_, ginsenoside Rg_1_, ginsenoside Re, ginsenoside Rb_1_, and ginsenoside Rd were analysed by HPLC as described in the text. NRR, Notoginseng Radix Et Rhizome; NRR-A, steamed NRR; NRR-B, NRR heated with sand; NRR-C, NRR fried with sesame oil.

**Table 2:** Contents of TG in raw and processed NRR treated steatotic L02 cells.

Sample	10 *μ*g/mL	20 *μ*g/mL	40 *μ*g/mL	80 *μ*g/mL	100 *μ*g/mL
NRR	0.40 ± 0.03^*∗∗∗*###^	0.41 ± 0.00^*∗∗∗*###^	0.48 ± 0.05^*∗∗∗*#^	0.51 ± 0.00^*∗∗∗*^	0.36 ± 0.03^*∗∗∗*###^
NRR-A	0.42 ± 0.00^*∗∗∗*###^	0.38 ± 0.01^*∗∗∗*###^	0.45 ± 0.02^*∗∗∗*###^	0.44 ± 0.01^*∗∗∗*###^	0.50 ± 0.03^*∗∗∗*^
NRR-B	0.44 ± 0.01^*∗∗∗*##^	0.41 ± 0.01^*∗∗∗*###^	0.39 ± 0.05^*∗∗∗*###^	0.39 ± 0.03^*∗∗∗*###^	0.38 ± 0.05^*∗∗∗*###^
NRR-C	0.44 ± 0.04^*∗∗∗*#^	0.48 ± 0.07^*∗∗∗*^	0.42 ± 0.04^*∗∗∗*^	0.55 ± 0.01^*∗∗∗*^	0.50 ± 0.02^*∗∗∗*^
Control group	0.25 ± 0.01^###^				
Model group	0.53 ± 0.01^*∗∗∗*^				
Lovastatin	0.31 ± 0.01^###^				
Fenofibrate	0.30 ± 0.01^###^				

Triglyceride (TG) contents were assayed by assay kits as described in the text. Values were mean ± SD (*n* = 3) and expressed in mmol/L. NRR, Notoginseng Radix Et Rhizome; NRR-A, steamed NRR; NRR-B, NRR heated with sand; NRR-C, NRR fried with sesame oil. NRR and its processed products were dissolved in 50% ethanol. *∗* indicates a significant difference compared with control group cells, and # indicates a significant difference compared with model group cells.

^*∗∗∗*^ *p* < 0.001. ^#^ *p* < 0.05. ^##^ *p* < 0.01. ^###^ *p* < 0.001.

**Table 3:** Contents of TC in raw and processed NRR treated steatotic L02 cells.

Sample	10 *μ*g/mL	20 *μ*g/mL	40 *μ*g/mL	80 *μ*g/mL	100 *μ*g/mL
NRR	0.12 ± 0.01^###^	0.14 ± 0.00^###^	0.13 ± 0.01^###^	0.13 ± 0.00^###^	0.13 ± 0.00^###^
NRR-A	0.16 ± 0.00^*∗*###^	0.18 ± 0.01^*∗∗∗*###^	0.13 ± 0.01^###^	0.15 ± 0.01^###^	0.14 ± 0.01^###^
NRR-B	0.14 ± 0.00^###^	0.15 ± 0.01^###^	0.17 ± 0.01^*∗∗∗*###^	0.16 ± 0.01^*∗*###^	0.16 ± 0.01^###^
NRR-C	0.13 ± 0.01^###^	0.18 ± 0.01^*∗∗∗*###^	0.13 ± 0.00^###^	0.18 ± 0.01^*∗∗∗*###^	0.18 ± 0.01^*∗∗∗*###^
Control group	0.13 ± 0.01^###^				
Model group	0.23 ± 0.00^*∗∗∗*^				
Lovastatin	0.12 ± 0.00^*∗∗∗*###^				
Fenofibrate	0.13 ± 0.01^*∗∗∗*###^				

Total cholesterol (TC) contents were assayed by assay kits as described in the text. Values were mean ± SD (*n* = 3) and expressed in mmol/L. NRR, Notoginseng Radix Et Rhizome; NRR-A, steamed NRR; NRR-B, NRR heated with sand; NRR-C, NRR fried with sesame oil. NRR and its processed products were dissolved in 50% ethanol. *∗* indicates a significant difference compared with control group cells, and # indicates a significant difference compared with model group cells.

^*∗*^ *p* < 0.05. ^*∗∗∗*^ *p* < 0.001. ^###^ *p* < 0.001.

**Table 4:** Contents of HDL-C in raw and processed NRR treated steatotic L02 cells.

Sample	10 *μ*g/mL	20 *μ*g/mL	40 *μ*g/mL	80 *μ*g/mL	100 *μ*g/mL
NRR	0.33 ± 0.03^*∗∗*##^	0.29 ± 0.02^*∗∗∗*^	0.30 ± 0.03^*∗∗∗*#^	0.32 ± 0.01^*∗∗∗*##^	0.33 ± 0.01^*∗∗*##^
NRR-A	0.28 ± 0.04^*∗∗∗*^	0.26 ± 0.04^*∗∗∗*^	0.23 ± 0.04^*∗∗∗*^	0.22 ± 0.01^*∗∗∗*^	0.32 ± 0.03^*∗∗∗*##^
NRR-B	0.41 ± 0.02^##^	0.33 ± 0.04^*∗∗*###^	0.31 ± 0.01^*∗∗∗*##^	0.33 ± 0.05^*∗∗*###^	0.31 ± 0.02^*∗∗∗*#^
NRR-C	0.27 ± 0.02^*∗∗∗*^	0.26 ± 0.02^*∗∗∗*^	0.26 ± 0.01^*∗∗∗*^	0.26 ± 0.02^*∗∗∗*^	0.23 ± 0.04^*∗∗∗*^
Control group	0.42 ± 0.02^###^				
Model group	0.22 ± 0.03^*∗∗∗*^				
Lovastatin	0.25 ± 0.31				
Fenofibrate	0.21 ± 0.03				

High-density lipoprotein cholesterol (HDL-C) contents were assayed by assay kits as described in the text. Values were mean ± SD (*n* = 3) and expressed in mmol/L. NRR, Notoginseng Radix Et Rhizome; NRR-A, steamed NRR; NRR-B, NRR heated with sand; NRR-C, NRR fried with sesame oil. NRR and its processed products were dissolved in 50% ethanol. *∗* indicates a significant difference compared with control group cells, and # indicates a significant difference compared with model group cells.

^*∗∗*^ *p* < 0.01. ^*∗∗∗*^ *p* < 0.001. ^#^ *p* < 0.05. ^##^ *p* < 0.01. ^###^ *p* < 0.001.

**Table 5:** Contents of LDL-C in raw and processed NRR treated steatotic L02 cells.

Sample	10 *μ*g/mL	20 *μ*g/mL	40 *μ*g/mL	80 *μ*g/mL	100 *μ*g/mL
NRR	1.48 ± 0.16^###^	1.43 ± 0.19^###^	1.51 ± 0.14^###^	1.33 ± 0.08^###^	1.56 ± 0.08^###^
NRR-A	1.63 ± 0.12^*∗∗*###^	1.87 ± 0.02^*∗∗∗*##^	1.47 ± 0.22^###^	1.47 ± 0.13^###^	1.73 ± 0.13^*∗∗∗*###^
NRR-B	1.19 ± 0.11^###^	1.27 ± 0.08^###^	1.32 ± 0.12^###^	1.40 ± 0.21^###^	1.21 ± 0.09^###^
NRR-C	1.70 ± 0.11^*∗∗*###^	1.75 ± 0.19^*∗∗*###^	1.81 ± 0.19^*∗∗*##^	1.83 ± 0.20^*∗∗*##^	2.32 ± 0.06^*∗∗∗*^
Control group	2.40 ± 0.12^###^				
Model group	1.23 ± 0.10^*∗∗∗*^				
Lovastatin	1.73 ± 0.12^###^				
Fenofibrate	1.54 ± 0.22^###^				

Low-density lipoprotein cholesterol (LDL-C) contents were assayed by assay kits as described in the text. Values were mean ± SD (*n* = 3) and expressed in mmol/L. NRR, Notoginseng Radix Et Rhizome; NRR-A, steamed NRR; NRR-B, NRR heated with sand; NRR-C, NRR fried with sesame oil. NRR and its processed products were dissolved in 50% ethanol. *∗* indicates a significant difference compared with control group cells, and # indicates a significant difference compared with model group cells.

^*∗∗*^ *p* < 0.01. ^*∗∗∗*^ *p* < 0.001. ^##^ *p* < 0.01. ^###^ *p* < 0.001.

**Table 6:** Contents of FFA in raw and processed NRR treated steatotic L02 cells.

Sample	10 *μ*g/mL	20 *μ*g/mL	40 *μ*g/mL	80 *μ*g/mL	100 *μ*g/L
NRR	713.91 ± 69.25^*∗∗∗*^	484.53 ± 25.77^###^	465.63 ± 41.66^###^	398.62 ± 47.67^###^	386.59 ± 15.46^###^
NRR-A	577.32 ± 32.19^*∗∗*#^	493.13 ± 82.53^###^	513.75 ± 47.89^##^	487.11 ± 27.57^##^	505.15 ± 14.58^##^
NRR-B	539.52 ± 24.36^*∗*###^	613.4 ± 62.96^*∗∗∗*#^	637.46 ± 61.06^*∗∗∗*#^	525.77 ± 5.15^*∗*###^	436.43 ± 31.5^###^
NRR-C	582.47 ± 96.57^*∗∗*##^	652.92 ± 12.97^*∗∗∗*^	673.54 ± 39.03^*∗∗∗*^	656.36 ± 75.99^*∗∗∗*^	817.87 ± 56.78^*∗∗∗*^
Control group	386.60 ± 5.15^###^				
Model group	762.89 ± 52.31^*∗∗∗*^				
Lovastatin	680.41 ± 8.93^*∗∗∗*^				
Fenofibrate	639.18 ± 94.47^*∗∗*^				

Free fatty acid (FFA) contents were assayed by assay kits as described in the text. Values were mean ± SD (*n* = 3) and expressed in mmol/L. NRR, Notoginseng Radix Et Rhizome; NRR-A, steamed NRR; NRR-B, NRR heated with sand; NRR-C, NRR fried with sesame oil. NRR and its processed products were dissolved in 50% ethanol. *∗* indicates a significant difference compared with control group cells, and # indicates a significant difference compared with model group cells.

^*∗*^ *p* < 0.05. ^*∗∗*^ *p* < 0.01. ^*∗∗∗*^ *p* < 0.001. ^#^ *p* < 0.05. ^##^ *p* < 0.01. ^###^ *p* < 0.001.
