# Supplementary material for: Lipid Regulation Effects of Raw and Processed Notoginseng Radix Et Rhizome on Steatotic Hepatocyte L02 Cell
Source: Biomed Res Int. 2016 Aug 24;2016:2919034. doi: 10.1155/2016/2919034 (PMC5013208; doi:10.1155/2016/2919034)
Supplement: Supplementary file 1 — The results of L02 cellular morphology by the inverted microscope were displayed in figure 1. 2. 3, and the lipid droplets accumulation in model group were more significantly than that in normal and positive group. Different concentrations of Raw and processed NRR and its triterpenoid saponins have different effects on cell morphology. [file 2919034.f1.docx]

**Supplementary Materials**

**
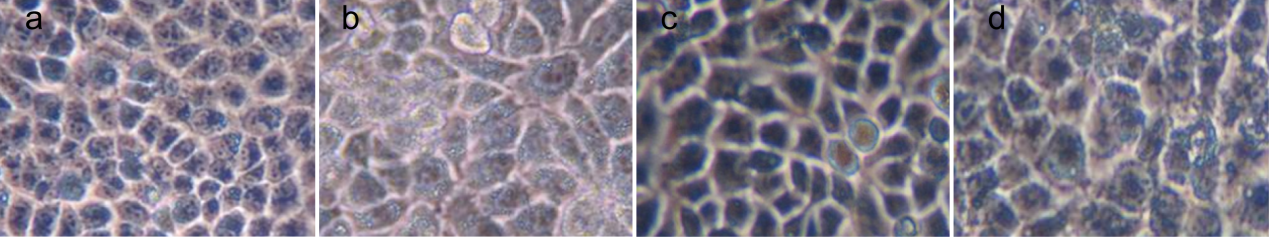
**

**Figure. 1.** Light microscopy of the LDs in L02 cells after different treatments.

(a) Control group, which were treated with 10% FBS-RPMI 1640 medium (b) Model group, which were treated with 5% fat emulsion-10% FBS-RPMI 1640 medium (c) Lovastatin, (d) Fenofibrate. All of these cells were treated with different medium at the concentration of 10ug/ml.


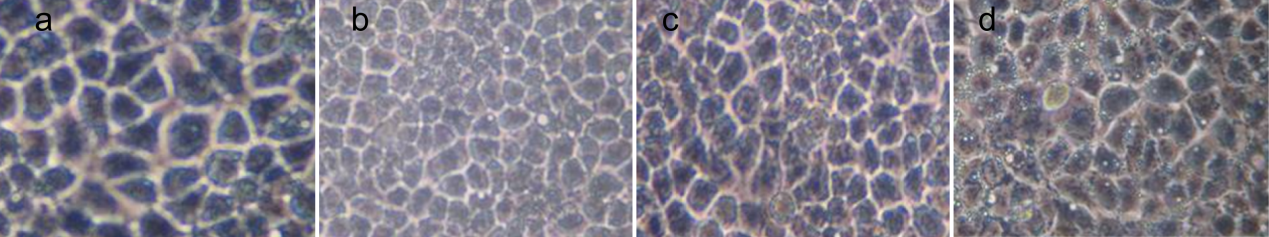


**Figure. 2 (a).** Light microscopy of the LDs in L02 cells after different treatments.

(a) NRR, (b) NRR-A, (c) NRR-B, (d) NRR-C. All of these cells were treated with raw and processed NRR at the concentration of 10ug/ml.


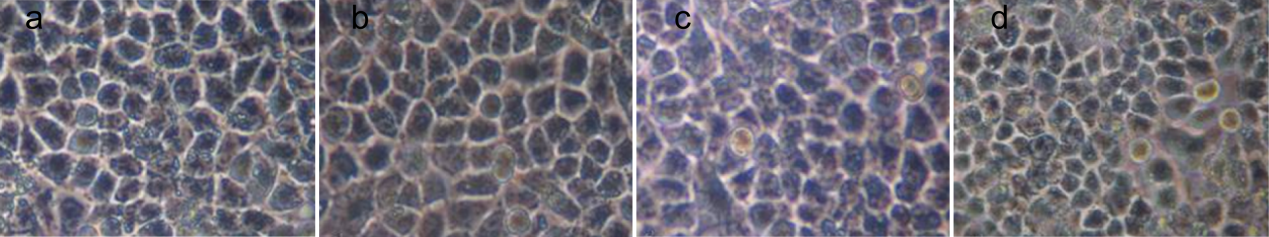


**Figure. 2 (b).** Light microscopy of the LDs in L02 cells after different treatments.

(a) NRR, (b) NRR-A, (c) NRR-B, (d) NRR-C. All of these cells were treated with raw and processed NRR at the concentration of 20ug/ml.


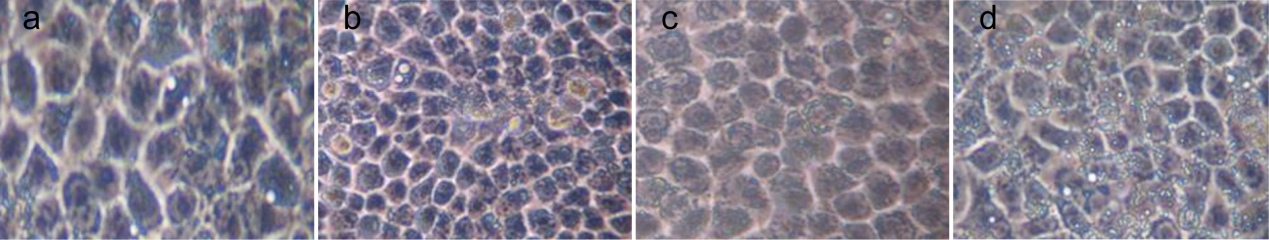


**Figure. 2 (c).** Light microscopy of the LDs in L02 cells after different treatments.

(a) NRR, (b) NRR-A, (c) NRR-B, (d) NRR-C. All of these cells were treated with raw and processed NRR at the concentration of 40ug/ml.


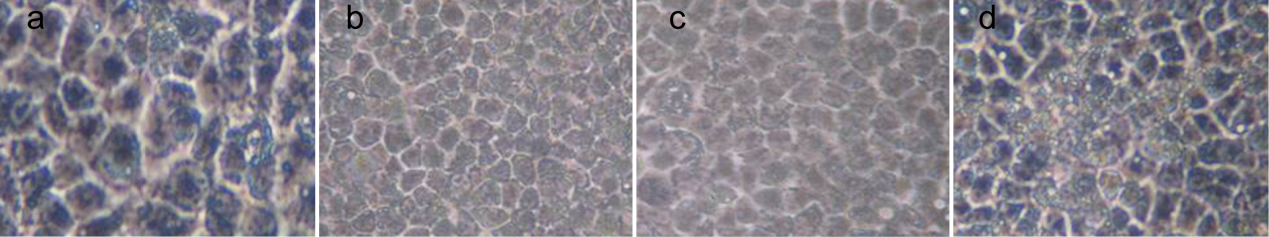


**Figure. 2 (d).** Light microscopy of the LDs in L02 cells after different treatments.

(a) NRR, (b) NRR-A, (c) NRR-B, (d) NRR-C. All of these cells were treated with raw and processed NRR at the concentration of 80ug/ml.


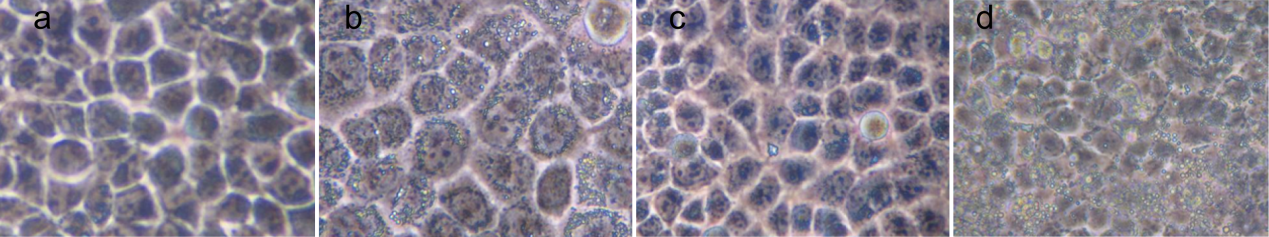


**Figure. 2 (e).** Light microscopy of the LDs in L02 cells after different treatments.

(a) NRR, (b) NRR-A, (c) NRR-B, (d) NRR-C. All of these cells were treated with raw and processed NRR at the concentration of 100ug/ml.


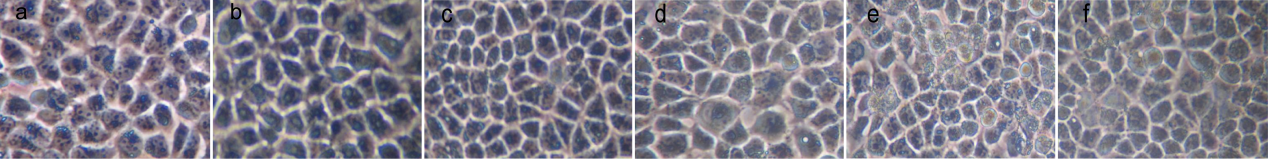


**Figure. 3 (a).** Light microscopy of the LDs in L02 cells after different treatments.

(a) PNS, Panax notoginsenosides, (b) notoginsenoside R_1_, (c) ginsenoside Rg_1_, (d) ginsenoside Rb_1_, (e) ginsenoside Re, (f) ginsenoside Rd. All of these cells were treated with saponin components at the concentration of 10ug/ml.


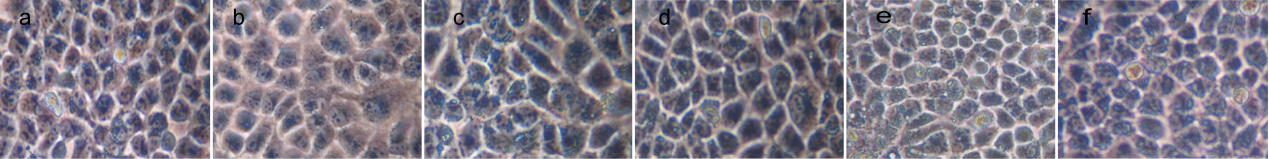


**Figure. 3 (b).** Light microscopy of the LDs in L02 cells after different treatments.

(a) PNS, Panax notoginsenosides, (b) notoginsenoside R_1_, (c) ginsenoside Rg_1_, (d) ginsenoside Rb_1_, (e) ginsenoside Re, (f) ginsenoside Rd. All of these cells were treated with saponin components at the concentration of 20ug/ml.


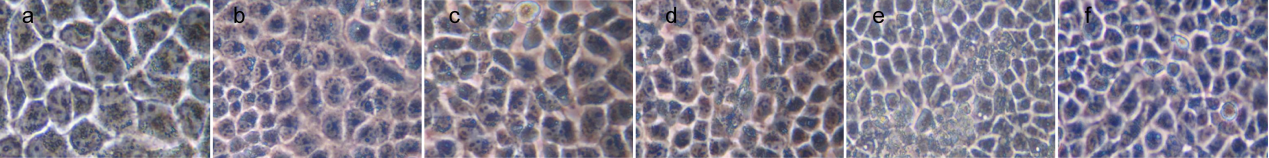


**Figure. 3 (c).** Light microscopy of the LDs in L02 cells after different treatments.

(a) PNS, Panax notoginsenosides, (b) notoginsenoside R_1_, (c) ginsenoside Rg_1_, (d) ginsenoside Rb_1_, (e) ginsenoside Re, (f) ginsenoside Rd. All of these cells were treated with saponin components at the concentration of 40ug/ml.
